# Concomitantly Diagnosed Disseminated *M kansasii* Infection and Hairy Cell Leukemia With Review of Pathophysiology

**DOI:** 10.1177/23247096241253343

**Published:** 2024-05-20

**Authors:** Daniel J. Stanton, Nadia Z. Quadri, Melinda B. Tanabe

**Affiliations:** 1 The University of Texas Medical Branch at Galveston, USA

**Keywords:** *Mycobacterium* kansasii, non-tuberculous mycobacterium, Hairy Cell Leukemia, immunocompromise, pathophysiology

## Abstract

The association between Hairy Cell Leukemia (HCL) and non-tuberculous mycobacterial infections (NTMs) is well described, most notably *Mycobacterium kansasii*. The exact pathophysiology is not known. We report a case of a 31-year-old male with concomitantly diagnosed HCL and disseminated *M kansasii* infection who presented with rash, pancytopenia, and bulky axillary lymphadenopathy. The *M kansasii* was initially diagnosed through use of cell-free DNA detection and confirmed by bone marrow and lymph node cultures. Hairy Cell Leukemia was diagnosed with peripheral flow cytometry and confirmed via the same bone marrow sample. His HCL was put into remission with a single course of cladribine and rituximab chemotherapy; however, his *M kansasii* infection persisted for 6 months despite aggressive antimicrobial and surgical therapy. It was finally controlled using high-dose rifampin in combination with azithromycin and ethambutol. This case highlights the known link between HCL and *M kansasii.* Furthermore, it hints at potential causes beyond chemotherapy-induced immunocompromise. Notable possibilities include HCL cells acting as sanctuary sites for *M kansasii* to evade the immune system, and subclinical *M kansasii* infections causing NLRP3 inflammasome overactivation to trigger the oncogenic transformation to HCL. More research into the pathophysiologic link between HCL and *M kansasii* infections would allow for more effective prevention, diagnosis, and treatment of these severe atypical infections which are the major cause of morbidity in the cladribine era of HCL treatment.

## Introduction

*Mycobacterium kansasii* complex is a non-tuberculosis mycobacterium (NTM) which is characterized as acid-fast bacilli (AFB) that can cause pulmonary infections and disseminated disease.^1^ *Mycobacterium kansasii* complex encompasses subtypes I to VI based on gene sequencing, with subtypes I and II being commonly pathogenic to humans.^2^ *Mycobacterium kansasii* is endemic to the United States, mostly in tap water as a reservoir with the highest rates of infection noted in Southern states such as Texas and Louisiana.^1^ The primary form of infection is cavitary pulmonary disease, but extrapulmonary manifestations have been described involving the lymph nodes, skin, musculoskeletal system, and urinary tract.^1^ Disseminated *M kansasii* is commonly reported in patients with other immunosuppressing diseases such as Human Immunodeficiency Virus infection and Hairy Cell Leukemia (HCL).^3-6^ However, little is known about the association between HCL and *M kansasii.* Hairy Cell Leukemia seemed to provide a gateway for opportunistic infections.^4,6^ In this report, we discuss a patient with concomitant newly diagnosed HCL and *M kansasii* infection.

## Case Report

A 31-year-old male from South Texas with no known past medical history, but a family history of hemochromatosis presented to our institution with a rash and febrile pancytopenia. Two weeks prior to admission, he was diagnosed with bacterial sinusitis after reporting headache, sinus pressure, and fever of 38.9°C, and he was given ciprofloxacin and ibuprofen. Patient then developed a non-pruritic, non-painful, confluent rash which began as a small lesion on his right hand. The rash spread rapidly up his arm to his trunk, neck, and other extremities, for which he was given prednisone. The patient worked as a construction worker, lived on a farm with his family, and denied any travel history or freshwater exposure.

**Figure 1.**
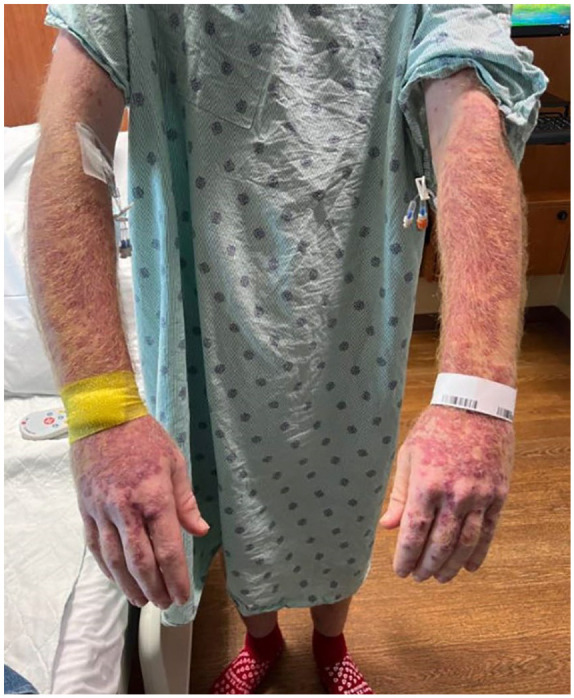
Image of patient’s rash.

On admission, patient had a blood pressure BP 122/83 mm Hg, pulse 118 bpm, respiratory rate 16/min, and a T 38.6°C. On physical exam, patient had a partially confluent erythematous maculopapular rash on all limbs with flaking scales (Figure 1). He denied weight loss, respiratory symptoms, nausea, vomiting, or diarrhea. Fist-sized 10 cm tender lymphadenopathy was present in the right axilla. Small tender supraclavicular lymphadenopathy and tender hepatosplenomegaly were also present. The rest of the examination was unremarkable. Laboratories showed: white blood cell count of 0.89 × 10^3^/mm^3^ (ref: 4500-11 000/mm^3^), hemoglobin 5.4 g/dL (male ref: 13.5-17.5 g/dL), platelets 51 000/mm^3^ (ref: 150 000-400 000/mm^3^), Na 130 mEq/L (ref: 136 to 145 mEq/L), aspartate transaminase (AST) 46 U/L (ref: 10-40 U/L), alanine transaminase (ALT) 67 U/L (ref: 10-40 U/L), alkaline phosphatase (ALP) 384 U/L (ref: 30 to 120 U/L), total bilirubin 1.7 mg/dL (ref: 0.3-1.0 mg/dL), and ferritin 1180 ng/mL (male ref: 12-300 ng/mL).

**Figure 2.**
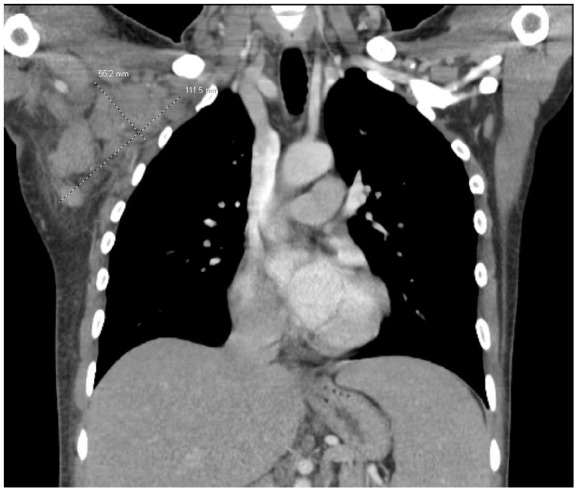
Computed tomography with contrast of patient’s thorax showing bulky lymphadenopathy in right axilla.

Computed tomography of the thorax and abdomen with contrast showed right ill-defined axillary lymphadenopathy measuring approximately 11 cm × 5.6 cm × 5.5 cm. This extended into the lateral subpectoralis and right supraclavicular lymph nodes. There was also splenomegaly measuring 20.5 cm × 13.2 cm × 7 cm present (Figure 2). Flow cytometry of peripheral blood revealed aberrant clonal B-cell population consistent with HCL, which was confirmed by bone marrow biopsy. He was also diagnosed with hemochromatosis via *HFE* gene mutation detection. Patient was started on cladribine and rituximab chemotherapy, followed by granulocyte colony stimulating factor. Patient responded well initially; however, 1 week later, he developed febrile neutropenia with T max 40°C. Standard infectious workup was non-contributory including bacterial, fungal, and AFB smears and cultures of the blood and bone marrow. Thus, 2 weeks after bone marrow culture was collected, cell-free microbe DNA testing was performed, resulting in *M kansasii* identification which took 10 days to result. The patient was started on azithromycin, rifampin, and ethambutol therapy. One week later, AFB cultures had growth from the bone marrow. Another week later, the microbes were able to be speciated and confirmed disseminated *M kansasii* infection. Finally, 8 weeks after collection of bone marrow, the sensitivity profile showed the *M kansasii* was rifampin and macrolide sensitive, both possessing minimum inhibitory concentrations of 0.12 µg/mL. No further sensitivities were initially tested per our reference laboratory’s protocol for *M kansasii*.

The patient’s course was complicated with persistent fevers and development of a new cold abscess. The abscess was initially in the right antecubital fossa with worsening axillary lymphadenopathy and sporotrichoid spread. Further computed tomography imaging of the thorax with contrast showed axillary lymph nodes with abscesses that were drained multiple times throughout his admission. Pathology from patient’s initial hand lesion showed granulomatous inflammation and AFB positive bacteria. This is the presumed source of his inoculation with *M kansasii* due to the sporotrichoid spread up his right arm into his axillary and neck lymph nodes. Given the persistent infection, amikacin was added to his treatment which caused him to defervesce. The patient was eventually discharged with home weight–based IV amikacin, and oral rifampin 600 mg daily, azithromycin 500 mg daily, and ethambutol 1200 mg daily.

The patient required several re-admissions for further incision and drainage of abscesses, which continued evolving to form new abscesses along his right upper extremity lymph node chain. He followed up closely in the infectious diseases and surgical oncology clinics for monitoring of abscess progression and medication adjustments, as well as with audiology and ophthalmology clinics to monitor for toxicities from his amikacin and ethambutol, respectively. His antimicrobials required frequent adjustment due to side effects. Development of persistent bilateral tinnitus and subclinical decrease in high-frequency sound perception resulted in discontinuing IV amikacin, and the damage appears to be permanent. A trial of linezolid was discontinued due to severe thrombocytopenia. Subsequent cultures were sent for expanded susceptibility testing which showed our isolate was resistant to trimethoprim/sulfamethoxazole, and intermediately resistant to tetracyclines and fluoroquinolones. Due to waning options for additional antimicrobials, the dose of rifampin was increased to 900 mg which drastically improved infection control by halting the development of new abscesses. Hairy Cell Leukemia remained in remission on repeat bone marrow biopsy at 4 months. Acid-fast bacilli cultures of abscesses continued to show growth until 6 months after diagnosis, where the most recent AFB culture was smear positive, but no organism grew. Currently, our patient will continue high-dose oral rifampin, azithromycin, and ethambutol for an additional 12 months.

## Discussion

This patient presented with simultaneous diagnosis of HCL and *M kansasii* infection, as evidenced by the axillary lymphadenopathy on admission, which was the primary site from which his AFB cultures remained positive over the course of his infection. The relationship between NTM and HCL has been loosely described, including the predilection for *M kansasii* disseminated infection compared to other NTMs.^3^ To the authors’ knowledge, no discrete mechanism has been identified to explain the association thus far. The existing postulate suggests that the immunocompromised state from chemotherapy allows for opportunistic infections with uncommon pathogens like *M kansasii.* This patient was also diagnosed with hemochromatosis, and this may have further increased his vulnerability to NTM infections.^7^ However, this does not specify how *M kansasii* is more commonly isolated than the more prevalent *Mycobacterium avium* complex in HCL patients. Furthermore, this theory does not explain this concomitant presentation.

Current research suggests that HCL cells may act as a sanctuary site for *M kansasii*.^8^ Hairy Cell Leukemia malignant cells are B cells which are uniquely undifferentiated to overexpress cytoskeletal components leading to hair cell projections.^7^ These hairy projections allow HCL cells to phagocytose bacteria.^7^ Each HCL cell may differ in their phagocytosis mechanism, which can occur at different time points in a single patient.^7^ *Mycobacterium kansasii* shares a significant amount of its genome with *Mycobacterium tuberculosis*.^2^ *Mycobacterium tuberculosis* is known to cause clinical disease by escaping from the phagosomes of macrophages and entering the cytosol using hyperspecialized antigens including Early Secretory Antigenic Target 6 (ESAT-6). It then alters cellular expression of numerous cytokines to form granulomas.^9,10^ Similarly, in patients with *Mycobacterium kansasii* infections, granulomas are frequently identified in the spleen, a major site of pathogenesis in HCL.^11,12^ After treatment with highly effective chemotherapy such as cladribine and rituximab, the intracellular NTMs are released in high quantities at once. This overwhelms the immune system of the already immunocompromised patient. Further research is required to confirm this theory, including the need for macrolide prophylaxis in HCL patients, as seen in HIV patients with *M kansasii* infection.^13^

Another potential link is the conjoined roles of the inflammasome in their pathophysiology. It has now been established that the NLRP3 inflammasome plays a key role in host immunity to *M kansasii* and other mycobacteria.^14^ Many blood cancers such as acute myeloid leukemia (AML) are associated with chronic activation of NLRP3.^15^ This pathway may also be involved in HCL through NLRP3 overactivation in splenic macrophages; thus, NTMs could lead to the development of HCL and other blood cancers.

The case above demonstrates the need for earlier diagnostic testing to prevent morbidity of disseminated NTM disease. As evidenced by prior case reports, the resultant delay in diagnosis via slow-growing cultures can lead to high morbidity and mortality.^4^ Our patient was rapidly diagnosed by cell-free DNA analysis which returned 1 week prior to the bone marrow culture growing an AFB organism, and 2 weeks prior to *M kansasii* being identified in culture. It aided in the rapid initiation of appropriate antimicrobial treatment, possibly sparing the patient morbidity. The cost to run commercially available cell-free DNA retails at approximately $2000 USD. The test is approved by clinical laboratory organizations for assisting in the diagnosis of pathogens in cases of pneumonia, culture negative infective endocarditis, and neutropenic fever. However, it is not yet reliably coverage by insurance companies. Its use in early diagnosis of microorganisms must be weighed against its cost, turnaround time, and implications in the individual providers’ empiric vs non-empiric treatment decision-making capacity.

The implementation of cladribine chemotherapy has significantly improved HCL prognosis; thus, the current main cause of morbidity is severe infections.^16^ This case highlights the importance of understanding the connection between HCL and *M kansasii* infection. This will help develop preventative measures, employ earlier advanced diagnostic methods, and treat infection more effectively. Future study includes the establishment causality between the 2 diseases, use of prophylactic macrolide or rifamycin therapy after chemotherapy for HCL, and a cost to benefit evaluation of routine use of cell-free DNA detection technology for neutropenic fever in patients with HCL.
